# Expert Perspectives on Diagnosis and Management of Vitamin B_12_ Deficiency in Adults^☆^

**DOI:** 10.1016/j.advnut.2026.100726

**Published:** 2026-08-25

**Authors:** Jean-Louis Guéant, David H Alpers, Lindsay H Allen, Emmanuel Andres, Angela M Devlin, Ari Green, Ralph Green, Rosa-Maria Guéant Rodriguez, Luciana Hannibal, Dominic Harrington, Joel B Mason, Andrew McCaddon, Joshua W Miller, Michelle Murphy, Ebba Nexo, Helene McNulty, Peter Julian Owen, Edward Quadros, David Rosenblatt, Helga Refsum, Wilemina Rietsema, Sally Stabler, Mary Ward, Chittaranjan Yajnik, Julia L Finkelstein

**Affiliations:** 1 Cornell Joan Klein Jacobs Center for Precision Nutrition and Health, Cornell University, Ithaca, NY, United States; 2 Department of Hepato-Gastroenterology, Reference Center of Inborn Errors of Metabolism, University Regional Hospital of Nancy, Vandœuvre-lès-Nancy, France; 3 DEVAH, Institute of Medical Research (Pôle BMS), University of Lorraine, Vandœuvre-lès-Nancy, France; 4 Department of Medicine, Washington University School of Medicine, St. Louis, MO, United States; 5 Department of Nutrition, USDA Agricultural Research Service Western Human Nutrition Research Center, Institute for Global Nutrition, University of California, Davis, Davis, CA, United States; 6 Service de Médecine Interne, Hôpital de Hautepierre, Hôpitaux Universitaires de Strasbourg, Strasbourg, France; 7 Department of Pediatrics, BC Children’s Hospital Research Institute, The University of British Columbia, Vancouver, BC, Canada; 8 Division of Neuroimmunology and Glial Biology, Department of Neurology, Department of Ophthalmology, Weill Institute of Neurosciences, University of California San Francisco, San Francisco, CA, United States; 9 School of Medicine, University of California, Davis, Davis, CA, United States; 10 Department of Biochemistry-Molecular Biology and Nutrition, Reference Center of Inborn Errors of Metabolism, University Regional Hospital of Nancy, Vandœuvre-lès-Nancy, France; 11 Department of General Pediatrics, Adolescent Medicine and Neonatology, Laboratory of Clinical Biochemistry and Metabolism, Faculty of Medicine, Medical Center, University of Freiburg, Freiburg, Germany; 12 Haematology, Guy's and St Thomas' NHS Foundation Trust, London, United Kingdom; 13 Faculty of Social and Life Sciences, Wrexham University, Wrexham, United Kingdom; 14 Jean Mayer USDA Human Nutrition Research Center on Aging, Tufts University School of Medicine, Friedman School of Nutritional Science & Policy, Tufts University, Boston, MA, United States; 15 Department of Nutritional Sciences, Rutgers University, New Brunswick, NJ, United States; 16 Unit of Preventive Medicine & Biostatistics, IISPV, CIBEROBN, Faculty of Medicine & Health Sciences, Universitat Rovira i Virgili, Reus, Spain; 17 Department of Clinical Biochemistry, Aarhus University Hospital, Aarhus, Denmark; 18 Nutrition Innovation Centre for Food and Health, Ulster University, Coleraine, Northern Ireland, United Kingdom; 19 Department of Trauma & Orthopaedics, Addenbrooke’s Hospital, Cambridge University Hospitals NHS Foundation Trust, Cambridge, United Kingdom; 20 SUNY Downstate Medical Center, Brooklyn, NY, United States; 21 Department of Human Genetics, McGill University, Montreal, QC, Canada; 22 Department of Pharmacology, University of Oxford, Oxford, United Kingdom; 23 Oxford Health NHS Foundation Trust, Oxford, United Kingdom; 24 Department of Medicine, University of Colorado Anschutz Medical Campus, Aurora, CO, United States; 25 Department of Endocrinology, King Edward Memorial Hospital, Pune, India; 26 Department of Nutrition, Institute of Basic Medical Sciences, Faculty of Medicine, University of Oslo, Oslo, Norway; 27 Cornell Cochrane Center, Cornell University, Ithaca, NY, United States; 28 Division of Epidemiology, Department of Population Health Sciences, Weill Cornell Medicine, New York, NY, United States; 29 Division of Nutrition, St. John's Research Institute, St. John's National Academy of Health Sciences, Bengaluru, India

**Keywords:** guidelines, vitamin B_12_ deficiency, folate, methylmalonic acid, homocysteine, autoimmune gastritis

## Abstract

There is no universally accepted definition of vitamin B_12_ deficiency despite the severity of its medical consequences if not properly diagnosed and treated. Vitamin B_12_ deficiency can arise either from inadequate dietary intake, or various causes of impaired absorption or availability. The latter include *Helicobacter pylori* and autoimmune gastritis, other causes of malabsorption, and drug side effects. Vitamin B_12_ deficiency can display diverse clinical signs and symptoms, including hematological, neurological, psychiatric, and thromboembolic manifestations. The minutes and conclusions of 3 workshops at the Federation of American Societies of Experimental Biology (FASEB) “B-vitamins and One-Carbon Metabolism” Scientific Research Conferences (2022 and 2024) and the 2025 International Conference on One-Carbon Metabolism, B-Vitamins and Homocysteine, the examination of the literature and the National Institute for Health and Care Excellence/DELPHI guidelines allowed us to identify gaps and issues on diagnostic criteria and definition, evaluation of causation, and treatment strategies of vitamin B_12_ deficiency across different health care settings in adults (>18 y). Our aim is to address these gaps and issues in global guidelines to be reviewed and disseminated by the International Union of Nutritional Sciences. We identified 4 main goals: *1*) definition of vitamin B_12_ deficiency; *2*) assessment of causality; *3*) differential diagnosis and evaluation of nutritional and metabolic comorbidities; and *4*) treatment and follow-up. The objective of such guidelines should be to reduce both underdiagnosis and overdiagnosis of vitamin B_12_ deficiency by integrating clinical biomarker assessment and considering cause-specific treatment pathways. This should ultimately improve patient outcomes worldwide and, by arriving at uniform diagnostic criteria, facilitate the conduct, interpretation, and integration of research observations to inform clinical guidelines.

**Statements of significance**

This work is the first effort to design globally applicable clinical guidelines for adult vitamin B_12_ deficiency that explicitly integrate clinical scoring and biomarker algorithms and systematic assessment of causality and comorbid nutritional states. Unlike existing regional guidelines, it proposes a combined use of the Grading of Recommendations Assessment, Development and Evaluation and DELPHI frameworks to develop global guidelines for the diagnosis and management of vitamin B_12_ deficiency.

## Introduction

Vitamin B_12_ [cobalamin (Cbl)] is a water-soluble vitamin. Its bioavailability depends on a complex cascade of absorption, transport, cellular uptake, and intracellular processing. Three proteins—intrinsic factor (IF), haptocorrin, and transcobalamin—and their respective membrane receptors facilitate its assimilation and transport [1,2]. Cbl serves as a cofactor for 2 mammalian enzymes: cytoplasmic methionine synthase (MS) and mitochondrial methylmalonyl-CoA mutase (MUT). Methylcobalamin is a cofactor of MS, which catalyzes a remethylation reaction in which the methyl group of 5-methyltetrahydrofolate is transferred to homocysteine (Hcy) to generate methionine and tetrahydrofolate. This fuels the methionine cycle and the cytoplasmic folate cycle, respectively. Adenosylcobalamin plays a key role in mitochondrial energy metabolism as a cofactor for MUT, which converts methylmalonyl-CoA to succinyl-CoA.

Mechanisms underlying the highly variable clinical presentation of vitamin B_12_ deficiency (Table 1 [[1], [2], [3], [4], [5], [6]]) are traditionally attributed to disruption of the folate cycle and consequent impairment of purine/pyrimidine biosynthesis, endogenous methionine and S-adenosyl-methionine production (*via* reduced MS activity), and disturbances of the anaplerotic pathway of the tricarboxylic acid cycle that catabolizes branched-chain amino acids, odd-chain fatty acids, and the cholesterol side chain. Over the past decade, data from cellular and animal models as well as inborn errors of vitamin B_12_ deficiency have highlighted key genomic and epigenomic pathophysiological mechanisms, including marked changes in gene expression affecting energy metabolism, endoplasmic reticulum stress, neurogenesis, neuroplasticity, and neurodevelopment [3,7].

**TABLE 1:** Clinical manifestations of vitamin B_12_ deficiency in adults [1‒3, 5‒7]

Hematological	Anemia, megaloblastic anemia, megaloblastosis, macrocytosis, normocytic anemia, leukocyte/platelet abnormalities, and reduced cell counts
Neurologic	Peripheral neuropathy, gait disturbance, ataxia, myelopathy, pyramidal/extrapyramidal syndromes, MRI brain atrophy
Cognitive	Cognitive decline, concentration difficulty, verbal communication deficits, memory loss
Psychiatric	Behavioral abnormalities, depression, delirium, hallucinations, dementia
Vascular	Thromboembolic disorders, venous thrombosis, stroke, and hypertension
Other	Glossitis, fatigue, sleep disorders, visual problems (optic neuropathy, nystagmus, scotoma, maculopathy, strabismus, blurred vision, optic nerve atrophy), infertility, recurrent pregnancy loss, hyperpigmentation
Manifestations of associated autoimmune disorders	Thyroiditis, vitiligo, diabetes

Vitamin B_12_ deficiency is a significant global medical and public health issue. Anemia is a major public health issue, for which vitamin B_12_ deficiency is one of the leading nutritional causes, along with iron and folate deficiencies. The main clinical manifestations of vitamin B_12_ deficiency are summarized in Table 1. Deficiencies arise from digestive malabsorption, inadequate dietary intake of animal source foods, iatrogenic causes, and, far more rarely, inborn errors of metabolism (Table 2 [2]). The condition can manifest with a wide spectrum of clinical signs and symptoms, including hematological abnormalities (macrocytosis to megaloblastic anemia), central and peripheral neurological disorders, psychiatric disturbances, and thromboembolic events [1,2,[4], [5], [6]].

**TABLE 2:** Dietary, digestive, and iatrogenic causes of vitamin B_12_ deficiency [2]

Risk factor/cause	Frequency	Mechanism	**Assessment (in addition to biomarkers of vitamin B_12_ status)**
Low intake of animal source foods	Frequent	Inadequate intake	Dietary history
Autoimmune gastritis (AIG/Biermer disease)	Relatively frequent	Impaired gastric acid and IF secretion	Endoscopy, fundic/antral biopsies, IF/parietal-cell antibodies, gastrin
Non-AIG gastritis	Relatively frequent	Impaired acid secretion, protein-bound vitamin B_12_ malabsorption	Endoscopy, histology
Intrinsic-factor (IF) deficiency (mutations in *CBLIF*)	Rare	Genetic intrinsic factor defect	Gene sequencing, family history
Imerslund-Gräsbeck disease (mutations in *CUBN*/*AMN*)	Rare	Genetic receptor defect	Gene sequencing
Adult-onset MM-ACHC disease	Rare	Genetic MM-ACHC defect	Gene sequencing
Bariatric surgery, gastrectomy, short bowel	Frequent	impaired intraluminal transport and ileal uptake	Surgical history, imaging
Small-Intestine Bacterial Overgrowth (SIBO)/blind-loop syndrome	Relatively frequent	Bacterial consumption of dietary vitamin B_12_	Hydrogen breath test
Tropical sprue/severe celiac disease	Relatively rare	Villous atrophy and impaired IF-mediated uptake of vitamin B_12_	Anti-tTG antibodies, endoscopy
Parasitic infestation	Relatively rare	Villous atrophy and vitamin B_12_ consumption	Stool analysis
Zollinger-Ellison syndrome	Rare	Acidic intestinal pH and impaired IF-mediated uptake of vitamin B_12_	Gastrin concentrations, imaging
Inflammatory bowel disease (Crohn's disease, ulcerative colitis)	Relatively frequent	Ileal mucosal injury and impaired IF-mediated uptake of vitamin B_12_	Endoscopy, imaging
Chronic radiation distal ileitis	Rare	Mucosal injury and impaired IF-mediated uptake of vitamin B_12_	Imaging
Pancreatic insufficiency	Rare	Impaired haptocorrin degradation	Lipase, steatorrhea assessment
CKD, ESRD on hemodialysis	Relatively rare	Loss of circulating holotranscobalamin	Diet and Supplement history
Chronic obstructive jaundice	Relatively rare	Impaired binding of vitamin B_12_ to intrinsic factor	Imaging, labs
Iatrogenic drug effects (e.g., proton pump inhibitors, metformin)	Frequent	Impaired uptake of vitamin B_12_/SIBO	Medication review

Abbreviations: AIG, autoimmune gastritis; CBLIF, gene of gastic intrinsic factor; CKD, chronic kidney disease; CUBN, gene of cubilin; AMN, gene of amnionless; ESRD, end stage renal disease; IF, intrinsic factor; MMACHC, metabolism of cobalamin associated C (human); tTG, transglutaminase.

### Gaps in existing guidelines and recommendations

The experts from our initiative participated in 2 workshops at organized at the FASEB (Federation of American Societies of Experimental Biology) B-vitamins and One-Carbon Metabolism Scientific Research Conferences (2022 and 2024) with support of NIH and 1 workshop organized in the 2025 International Conference on One-Carbon Metabolism, B Vitamins and Homocysteine in Nancy, France, with the support of the French Society of Nutrition and cochaired by the president of the Nutrition Society of Great Britain and Ireland.

The minutes and conclusions of these workshops and the examination of the literature and the National Institute for Health and Care Excellence (NICE)/Delphi guidelines allowed us to identify gaps and issues which were presented to the International Union of Nutritional Sciences with the perspective to inform global guidelines on diagnostic criteria and the definition, evaluation of causation, and treatment strategies of vitamin B_12_ deficiency. The Delphi method to produce guidelines is a structured communication process that uses multiple rounds of questionnaires to build a consensus among a panel of experts. Guidelines addressing rare forms of vitamin B_12_ deficiency caused by mutations in genes of the vitamin B_12_ pathway have been produced by the European Network and Registry for Homocystinurias and Methylation Defects and will not be considered [8].

Despite the high prevalence of vitamin B_12_ deficiency among world regions and the severity of its medical consequences, there is no universally accepted definition of vitamin B_12_ deficiency or consensus on its diagnosis and management. Because none of the manifestations are pathognomonic, the diagnosis of vitamin B_12_ deficiency must rely on a combination of clinical acumen and laboratory markers. Biomarkers have been examined in the Centers for Disease Control and Prevention NHANES Roundtable and NIH Biomarkers of Nutrition for Development (BOND) initiatives [1,9,10], but these initiatives did not establish which biomarkers, cutoffs, or which measurement or combination of measurements provides the most valuable diagnostic information. Two recent guidelines on the diagnosis, management, and treatment of vitamin B_12_ deficiency—the United Kingdom NICE guideline and a Delphiexpert consensus from Germany and neighboring European countries—have also produced contrasting conclusions [11,12]. We aimed to identify the gaps and issues to be addressed for preparing global guidelines for review and dissemination through the International Union of Nutritional Sciences.

In the above-mentioned workshops, we identified issues related to the diagnosis of vitamin B_12_ deficiency, harmonization of cutoffs for vitamin B_12_ analyses according to commercial methods, and the respective use of other first- and second-line metabolic markers in the diagnostic process, depending on the clinical context. In the absence of universal screening, the first step toward identifying vitamin B_12_ deficiency depends on the judgment of a physician who must first suspect vitamin B_12_ deficiency and then request appropriate testing to confirm or rule out the diagnosis. This step is not easy, and the outcome is not always binary. One of the most perplexing problems in the clinical setting is interpreting mildly to moderately low serum vitamin B_12_ concentrations without further information. Current commercial assays for vitamin B_12_ (and folate) perform poorly in inter-laboratory surveys, leading to result variation and divergent interpretations, with many low vitamin B_12_ values being false positives. Serum methylmalonic acid (MMA) and plasma Hcy are useful metabolic biomarkers of vitamin B_12_ deficiency. MMA is the most specific indicator of vitamin B_12_ deficiency, whereas serum Hcy is also a sensitive indicator of folate deficiency. Deficiencies in both vitamin B_12_ and folate cause identical megaloblastic anemia; accurate differentiation is essential to avoid inappropriate folic acid supplementation in vitamin B_12_ deficiency and needless vitamin therapy in primary bone-marrow disorders. In routine practice, Hcy and MMA pose challenges unless concentrations are markedly abnormal. Hyperhomocysteinemia may also be due to alcohol use disorder, antifolate drugs, and pathologic glomerular filtration.

### General comments on the NICE and DELPHI guidelines

The NICE and DELPHI guidelines are based on exhaustive literature analyses (Table 3 [11,12]). Their conclusions illustrated the need to develop guidelines according to clinical context and practicality among countries. Both guidelines underscored evidence-based, context-specific recommendations. A brief summary of their comparison is given in Table 3.

**TABLE 3:** General comments on the National Institute for Health and Care Excellence [11] and Delphi [12] guidelines

Aspect	NICE (United Kingdom)	Delphi (Europe)
Scope and applicability	Developed primarily for the United Kingdom. NICE recommendations may be applied to other health-care systems that have comparable access to prescribing, biomarkers, endoscopy, and therapy.	Applicability in European countries with no prescription limitation of biomarkers, endoscopy, and therapy.
Methodology	Expert panel, including clinicians, researchers, and patients.	Expert opinions were captured *via* clearly defined questions; consensus was expressed as percentages (%) with confidence intervals.
Evidence appraisal	Systematic review; GRADE to evaluate the certainty of evidence	Extracted data from the literature across a broad set of items. Emphasizes expert opinions and clinical context.
Cut-off for vitamin B_12_	Threshold for vitamin B_12_ deficiency (130 pmol/L) is lower than those used internationally (e.g., WHO)	—
First-line tests	Both serum vitamin B_12_ and holotranscobalamin (holoTC) are acceptable as first-line (no hierarchy)	Serum vitamin B_12_ or holoTC
Second-line markers	Methylmalonic acid (MMA) only when initial results are indeterminate. Homocysteine (Hcy) receives limited attention. The panel does not suggest measuring folate or Hcy to rule out folate deficiency.	Favors MMA as the primary metabolic marker, but does not exclude Hcy: “Measurement of plasma MMA (or Hcy if MMA is unavailable) aids in diagnosing vitamin B_12_ deficiency.” Both markers are valuable when the clinical picture is unclear.
Additional testing for diagnosis	Recommendations for definitive diagnosis and follow-up are sparse; the guideline offers little guidance on endoscopic evaluation or on assessing immunologic or inflammatory markers of gastrointestinal disease.	Diagnosis of AIG is supported by detecting serum antibodies against parietal cells or intrinsic factor; endoscopic confirmation is not emphasized. Assessment of iron and folate status is incorporated into the diagnostic algorithm.
Treatment	Parenteral vitamin B_12_ is advised for autoimmune gastritis (AIG), total gastrectomy, or extensive terminal-ileal resection. For other malabsorption causes, either parenteral or high-dose oral supplementation (1 mg/d) is acceptable.	No clear preference for parenteral compared with oral therapy; both routes are considered acceptable depending on the underlying cause.

Abbreviations: GRADE, Grading of Recommendations Assessment, Development and Evaluation; NICE, National Institute for Health and Care Excellence; The Delphi method to produce guidelines is a structured communication process that uses multiple rounds of questionnaires to build a consensus among a panel of experts.

#### Besides some contrasting conclusions of these guidelines, key issues remain to be addressed

- Universal screening based on a clear definition of vitamin B_12_ deficiency is challenging as reliance on physician suspicion creates variability [1,13].
- A validated approach is needed to weigh clinical manifestations attributable to vitamin B_12_ deficiency.
- Use of adapted biomarkers and assessment for causality are underscored. Standardized cutoffs for holotranscobalamin (holoTC), MMA, and Hcy differ in the literature and among world regions. In previous guidelines, serum vitamin B_12_ and/or holoTC are considered first-line biomarkers [11,12]. However, vitamin B_12_ deficiency may exist despite a normal blood vitamin B_12_ concentrations. Therefore, there is a need for a systematic review and meta-analysis of biomarkers to design clear recommendations for use of biomarkers in diagnosis and management of vitamin B_12_ deficiency.
- Recommendations for assessing vitamin B_12_ absorption are limited, and no clinically validated testing for vitamin B_12_ malabsorption has been established.
- Integration of folate status and Hcy is limited in the NICE guidlines.

### Goals to be achieved for development of vitamin B_12_ deficiency guidelines

Our proposal for the development of these guidelines includes 4 objectives for recommendations on: *1*) a clear definition of vitamin B_12_ deficiency, *2*) the assessment of causality, *3*) the assessment of differential diagnoses and evaluation of nutritional and metabolic comorbidities, and *4*) the treatment and follow-up.

#### Definition of vitamin B_12_ deficiency

Establishing a clear definition of vitamin B_12_ deficiency is the first goal to be addressed.

It relies on *1*) the spectrum of clinical manifestations, *2*) the clinical and nutritional context, and *3*) the results of appropriate biomarker combinations, which will be interpreted according to their specific limitations and the metabolic and nutritional context. The objective is to define the cases that need initial pharmacologic treatment with vitamin B_12_.

##### General considerations of clinical context, signs and/or symptoms, and biomarkers

A key issue to consider is the clinical context in which a physician suspects a *possible* vitamin B_12_ deficiency, as, in practice, she/he is faced with a wide spectrum of non-specific clinical symptoms. The definition should take into account *1*) the clinical context, *2*) signs and/or symptoms observed by a proper report of outcomes and clinical examination of the patient to assess hematological, neurological, cognitive, dermatological, mucosal (tongue), and ophthalmological signs, and *3*) a proper prescription of first-line biomarkers of vitamin B_12_ deficiency that excludes folate deficiency, as some clinical outcomes are similar in both deficiencies [9,14]. The list of manifestations that can be reasonably allocated to vitamin B_12_ deficiency should first be considered by attributing a specific weight to manifestations. To achieve this goal, a careful evaluation of the frequency of outcomes in published patient series is needed, through a systematic review with meta-analysis similar to that recently used for inherited disorders [15]. For example, fatigue alone cannot be considered an initial manifestation in the absence of any other manifestations. In contrast, fatigue associated with peripheral neuropathy and macrocytosis, even in the absence of anemia, does suggest vitamin B_12_ deficiency. Likewise, a vitamin B_12_-deficient state defined solely on the basis of biomarker concentrations should be interpreted with caution.

##### The medical situations in which the assessment is performed should be considered in the criteria for defining vitamin B_12_ deficiency

We propose to categorize the landscape of situations that will strengthen the plausibility of vitamin B_12_ deficiency and be considered in the treatment and follow-up guidelines. A preliminary categorization is proposed in Table 4. Patients in categories A and B should be considered in the guidelines, and complementary advisory comments are warranted for categories C and D.

**TABLE 4:** Categories of clinical presentations of vitamin B_12_ deficiency and deficits

A. Patients with signs and/or symptoms of deficiency and a known etiology for vitamin B_12_ deficiency. For example, presentations type 1‒6	1. Autoimmune gastritis diagnosed by endoscopy 2. Atrophic gastritis of any other etiology, diagnosed by endoscopy 3. Resection of the gastric body or total gastrectomy 4. Short bowel syndrome with loss of terminal ileum 5. Severe celiac disease and other chronic intestinal diseases causing injury to the mucosa with evidence for extensive small bowel involvement and/or impaired intrinsic factor-mediated ileal uptake of vitamin B12 (e.g., radiation enteritis) 6. Lack of intake of animal source foods, no supplements for >6‒12 mo
B. Patients with signs and symptoms and confirmed vitamin B_12_ deficiency biomarkers but without clear causation	• Hematologic signs and symptoms • Neurologic/psychiatric/fatigue signs and symptoms • Other organs (e.g., skin, tongue) signs and symptoms, responding to vitamin B_12_
C. Patients with type 1‒5 from A with abnormal biomarkers (serum vitamin B_12_ + MMA and Hcy, or holoTC) but without signs and/or symptoms	
D. Patients with symptoms and uncertain vitamin B_12_ deficiency biomarkers (for example, isolated low serum vitamin B_12_ and weak increase of 1 of the metabolic biomarkers Hcy and MMA)	
E. Patients with vitamin B_12_ deficit defined by no clinical outcomes and abnormal biomarker concentrations (will not be considered in the guidelines)	

Abbreviations: Hcy, homocysteine; holoTC, holotranscobalamin; MMA, methylmalonic acid.

A first-line diagnosis can be made by a general practitioner (GP), according to classical/frequent manifestations and clinically evocative context (TABLE 2, TABLE 4), such as digestive diseases known to cause malabsorption, and digestive surgery such as bariatric surgery, gastrectomy, and ileectomy [2]. She/he should request first- and second-line biomarkers to conform with the requirements needed for performing the diagnosis, but in fact, many GPs often limit their request to serum vitamin B_12_ or, in some places, holoTC. Except when an evident cause, such as veganism/vegetarianism, is present, additional exploration for causality should be performed to clarify the relationship of the clinical manifestations to true vitamin B_12_ deficiency, including an in-depth clinical examination and biomarker abnormalities. With this approach, the patient should be referred to a specialist in internal medicine, gastroenterology, hematology, or neurology, or to a practitioner at a metabolic disorder referral center [2,4]. When primary evidence for a cause of vitamin B_12_ deficiency is lacking, assessing causality is mandatory. This will rely on the expertise of the specialist, who should consider all other causes that may explain the spectrum of the clinical manifestations and not orient the whole assessment only to vitamin B_12_ deficiency etiology.

Depending on the initial symptoms, patients with vitamin B_12_ deficiency may present to various specialists, including those in neurology, hematology, gastroenterology, internal medicine, pediatrics, or vascular medicine. In such circumstances, specialists often exclude all the usual causes of the clinical presentation but may ignore vitamin B_12_ deficiency as a possible cause. This is particularly the case in vascular medicine [4,13]; thromboembolic disorders are often inaugural manifestations of autoimmune gastritis (AIG) with vitamin B_12_ deficiency and dramatic hyperhomocysteinemia, even in the absence of folate deficiency [4].

Specialists may notice hallmarks indicative of vitamin B_12_ deficiency, such as low serum vitamin B_12_, increased Hcy, and/or macrocytosis. They often address this issue by treating the deficiency but without considering its causality.

In practice, causality should not be ignored, including by referring the patient to a gastroenterology or clinical nutrition clinic for assessment of causality [2,4,16,17]. When patients are directed to such a clinic, they may have only been assessed for classical etiologies of the predominant manifestations, without considering other signs and symptoms possibly attributable to a vitamin B_12_ deficiency. The referral center clinician will consider the whole spectrum of vitamin B_12_ deficiency manifestations and, if the data confirm a deficiency, will investigate its causality and provide the appropriate treatment [4,16,17].

#### Assessment of causality

Causes of vitamin B_12_ deficiency comprise 3 groups: inadequate diets, digestive disorders resulting in vitamin B_12_ malabsorption, and iatrogenic causes [[1], [2], [3]]. The guidelines should consider the synergistic association of 2 or more causes belonging to these 3 groups. This part is mainly managed by gastroenterologists and clinical nutritionists. A proper stepwise work-up should include a clinical interview and examination, serologic autoantibodies, endoscopy, imaging, and genetic testing when indicated [2,14,16,17].

Dietary inadequacy should be considered in the whole population and in conditions with increased risk due to increased needs (e.g., pregnancy) or decreased uptake/bioavailability (e.g., aging). The main causes of malabsorption are listed in Table 2. Among them, special attention should be paid to the diagnosis of AIG, other gastritis, bariatric surgery, and inflammatory bowel disease [2,17]. The guidelines should propose an assessment that includes endoscopic examination and, if needed, video capsule, entero-TDM (tomodensitometry), breath testing, and biomarkers of malabsorption related to gastric and small bowel causes [2,17]. If a strong familial context exists, genetic assessment will be considered to investigate the contribution of pathogenic variants from genes involved in the intraluminal and ileal steps of vitamin B_12_ absorption and predisposition to autoimmune digestive disorders according to the identified cause (Table 2).

#### Differential diagnosis and evaluation of nutritional and metabolic comorbidities

Systematic screening for concurrent deficiencies (e.g., iron, folate, vitamin B_6_, vitamin B_2_, vitamin D) that may lead to observed hematologic or neurologic findings is needed for differential diagnosis of vitamin B_12_ deficiency. A third goal to be considered is therefore to assess nutritional and metabolic comorbidities related to causality. These can influence the clinical spectrum, biomarker assessment, nutritional and metabolic presentation, and treatment.

Apart from inherited disorders, most causes of malabsorption are not vitamin B_12_ specific and produce deficiencies in other micronutrients that can contribute to the clinical spectrum of manifestations and influence the interpretation of biomarkers [2,17]. Vegetarianism, veganism, and digestive disorders produce a spectrum of clinical and bioclinical manifestations that also reflect deficiencies or deficits in other micronutrients. For example, iron deficiency, AIG, bariatric surgery, and inflammatory bowel disease. It influences the hematological manifestations, including anemia severity, mean corpuscular volume of red blood cells, and the patient’s response to treatment [2,17]. Folate and other B-vitamin deficiencies may also cause anemia and neurologic outcomes, and this must be considered in the assessment of the etiologic diagnosis [[18], [19], [20]]. Guidelines are needed for assessing these comorbidities and/or possible alternate diagnoses and for the proper interpretation of biomarkers.

#### Treatment and follow-up

Therapeutic choice (e.g., standard-dose oral, high-dose oral, intramuscular, or subcutaneous) for vitamin B_12_ deficiency should be guided by cause, severity, and patient preference. Defined monitoring protocols should be tailored to etiology (e.g., periodic endoscopy for AIG, MRI for neurologic sequelae, MMA/Hcy trends).

##### Treatment

The most appropriate therapy should be based on the cause, symptom severity, short-term effect on nutritional and metabolic parameters, and medium-term effect on clinical manifestations. It is important to note that some clinical manifestations, particularly neurologic ones, may persist despite the correction of nutritional and metabolic parameters.

In contrast to the NICE and DELPHI guidelines, further attention is needed for a 2-step treatment: restoration of the body content of vitamin B_12_ and maintenance of body stores of the vitamin [11,12]. There is considerable literature on different dosage modalities and routes of administration proposed for treating vitamin B_12_ deficiency depending on the presence or absence of malabsorption [21]. The choice of route of administration may depend on the clinical presentation and the causal diagnosis. The preferred route and the criteria for assessing its effectiveness, both clinical and using biomarkers, are subject to debate. Oral administration predominates in some countries. Nasal administration has also been proposed [21]. Patients with a deficiency due to inadequate dietary intake generally do not have vitamin B_12_ malabsorption. In this case, oral supplementation is appropriate at a dosage close to physiologic requirements, once body stores are repleted [21]. Parenteral treatment (intramuscular or subcutaneous) is predominantly used in more severe forms of malabsorption and/or when oral treatment has proven ineffective [4,21]. Oral treatment is effective at high doses, >500 *μ*g/d, with a commonly used dose of 1000 *μ*g/d. Absorption is dose-dependent and involves a passive diffusion mechanism independent of the absorption mediated by gastric IF and the ileum Cubam receptor complex [2,21]. Some reference centers offer a precision nutrition approach based on the etiologic diagnosis, assessment of efficacy, and patient preferences [4]. In some cases, treatment combining parenteral and enteral routes is used when monthly parenteral treatment is insufficient to correct metabolic and hematological markers [4]. Vitamin B_12_ has no established upper intake level. It should be noted that parenteral treatment can rarely produce pseudoallergic manifestations, mainly pruritus and urticaria [22]. Reciprocally, a higher prevalence of gastrointestinal symptoms and vitamin B_12_ deficiency is observed in patients with chronic urticaria, making causality difficult to establish [23]. Besides the direct adverse effects of parenteral vitamin B_12_, there is a debate in the literature regarding high total serum/plasma vitamin B_12_ concentrations. Elevated serum vitamin B_12_ concentrations have been associated with cardiovascular diseases, cancer, and increased all-cause mortality risk [[24], [25], [26]]. This points out the need for guidance on clinical manifestations, and the development of standardized cutoffs for vitamin B_12_.

##### Guidelines for monitoring the follow-up of vitamin B_12_ deficiency according to their cause

Guidelines should consider the treatment options, according to compliance with oral *compared with* parenteral therapy, the criteria to be considered for evaluation of treatment efficacy (clinical and biochemical), and the management of patient groups at risk for developing vitamin B_12_ deficiency depending on the clinical context.

We propose 3 prevalent examples, AIG, Crohn’s disease, and bariatric surgery, to illustrate that monitoring is highly dependent on the cause of vitamin B_12_ deficiency.

Monitoring modalities for AIG vary considerably, depending on contrasted guidelines among countries, in particular for the use of endoscopy and clinical biomarkers. They must consider the correction of the deficiency but also comorbidities and risk of associated pathologies, particularly gastric and neuroendocrine cancers.

Monitoring vitamin B_12_ deficiency in Crohn’s disease is part of the recommendations by the American Gastroenterological Association for the clinical practice update on diet and nutritional therapies in patients with inflammatory bowel disease [27]. It is part of the broader nutritional monitoring of patients, who often also have deficiencies in other micronutrients, including iron and vitamin D, where the inflammatory context makes it more difficult to interpret biomarkers.

Monitoring patients after bariatric surgery is also the subject of numerous studies and recommendations. Vitamin B_12_ is the main micronutrient concerned, along with iron and vitamin D [[28], [29], [30], [31]].

The proposed guidelines will focus on adults (>18 y), and will not include pregnant or lactating women or children, which will be addressed in a separate effort. Monitoring during pregnancy is a specific task with several aspects. Biomarker concentrations differ between the 3 trimesters of pregnancy, with decreased concentrations of total vitamin B_12_, maintenance of holoTC concentrations, and increased concentrations of Hcy and MMA in the last trimester, without established thresholds for diagnosis [[32], [33], [34], [35]]. The stratification between vitamin B_12_ deficiency by biomarkers and true clinical deficiency may be difficult according to the clinical landscape. Vitamin B_12_ deficiency during pregnancy may increase risk of abnormal neurodevelopment with cognitive consequences in newborns and infants, and may be associated with risk of neural tube defects [[36], [37], [38]]. It may increase the subsequent risk of diabetes in children born to mothers with vitamin B_12_ deficiency at the first trimester of life [39,40]. It puts the mother and the infant at risk of deficiency during lactation when the mother has a pre-existing deficit in vitamin B_12_ before pregnancy [41]. “Treatment compared with prevention” is a point to be addressed according to public health criteria that vary among countries.

### Overview of the proposed protocol

We plan to register this protocol in PROSPERO. Briefly, we plan to combine the use established methods including Grading of Recommendations Assessment, Development and Evaluation (GRADE) and DELPHI frameworks to develop global guidelines for the diagnosis and management of vitamin B_12_ deficiency. This approach will preserve evidence-based principles in GRADE while addressing uncertainty through expert consensus with DELPHI to develop a rigorous, implementable, and globally applicable framework. Indeed, as explained in sections “General comments on the NICE and DELPHI guidelines” and “Goals to be achieved for producing vitamin B_12_ deficiency guidelines”, vitamin B_12_ deficiency poses diagnostic and therapeutic challenges because of limited evidence, lack of gold standard for its definition, inconsistent laboratory standards, and limitations in the use of biomarkers and search for causality and practice variability. An integrated DELPHI–GRADE methodology will merge evidence-based rigor (GRADE) with structured expert consensus (DELPHI) to address these gaps effectively.

We will adhere to standard protocols already used for the development of guidelines on inherited disorders of vitamin B_12_ metabolism [8]. The expert committee will be cochaired by J-LG and JLF, bringing together expertise in clinical diagnosis and biomarker management of vitamin B_12_ deficiency with rigorous methods in evidence synthesis and translation.

We will use the GRADE framework to include and update the research publications considered in the 2 previous NICE and DELPHI guidelines. This will include a systematic review of the evidence with GRADE assessments and transparent evidence-to-decision (EtD) processes. To complement this, we will integrate DELPHI methods to formalize consensus on ambiguous issues, reduce bias, and harmonize international standards. For full transparency, each recommendation will be supported by the final vote and any additional comments of the experts defined in the DELPHI framework, which will be presented in specific tables. Discrepancies and any disagreements will be resolved by an established 10-member external adjudication committee (chair: DHA). Because global recommendations must be tailored to regional clinical and health care contexts, we will ensure that the landscape of situations is considered, including them, particularly regarding the diversity of resource-limited settings and health care systems.

Conflicts of interest will be declared a priori as part of the screening process for experts, using a similar approach as Cochrane and WHO (and society journals, e.g., American Society for Nutrition).

The multidisciplinary, internationally representative participants will ensure diverse input, with representation of the main medical specialties needed (2 general practitioners, 2 gastroenterologists, 2 hematologists, 1 neurologist, 1 specialist in internal medicine, 2 specialists in clinical nutrition, 2 medical clinical chemists, and experts in biochemistry, nutrition, and epidemiology). Consensus thresholds will be set at ≥80% (strong consensus), 67–79% (moderate consensus), or <67% (no consensus). Statements without consensus will be flagged as conditional recommendations or research priorities. Special attention will be paid to discrepant conclusions from previous NICE and DELPHI guidelines. For example, in symptomatic patients with low-normal vitamin B_12_ concentrations, the NICE committee reported that there was weak evidence for MMA testing, whereas the DELPHI achieved 84% consensus to recommend confirmatory MMA testing for vitamin B_12_ concentrations between 148 pmol/L and 260 pmol/L. In the DELPHI guidelines, this final recommendation was conditional, characterized by low-certainty of evidence but strong consensus.

The proposed 4-phase framework includes:

1. **Scoping (GRADE):** Define clinical questions using PICO (Population, Intervention, Comparison, and Outcome) framework.
2. **Evidence review:** Conduct 2 systematic reviews with GRADE assessments and meta-analyses–one on clinical manifestations of vitamin B_12_ deficiency and a second on vitamin B_12_ biomarkers.
3. **DELPHI consensus:** Resolve gaps in diagnostic criteria (e.g., scoring the specific weight of clinical manifestations and symptoms, clinical context, cutoffs for serum total vitamin B_12_ and other biomarkers, MMA and Hcy use and interpretation), functional deficiency, causality assessment, and treatment thresholds *via* iterative expert feedback.
4. **Integration with EtD:** Combine evidence and consensus, prioritizing high-certainty evidence while using DELPHI for areas of uncertainty.

The proposed methods will offer transparency, harmonization, and adaptability, reducing practice variation in subclinical deficiency, asymptomatic cases, and long-term therapy. The final guidelines will include definitions, diagnostic algorithms, causation frameworks, treatment strategies, monitoring protocols, and special population considerations.

We will conduct structured systematic searches of the literature and cross-reference with the updated list of articles that were selected in the 2 previous NICE and DELPHI guidelines, completed by other articles with inclusion criteria restricted to adults (>18 y) and with documented vitamin B_12_ deficiency and reporting clinical outcomes and biomarker data. Vitamin B_12_ deficiency in pregnancy and pediatric populations will be addressed in separate guidelines. Selection criteria of literature for study design will include randomized clinical trials, cohort studies, systematic reviews, meta-analyses, and selected case reports for rare manifestations. Exclusion criteria will be studies of participants aged <18 y, pregnant or lactating women, narrative reviews, animal/in-vitro studies, editorials, and reports focusing solely on inherited vitamin B_12_ disorders (except when relevant to malabsorption).

The expected outputs will include the development of guidelines for:

• Optimal definition of the clinical context of vitamin B_12_ deficiency.
• Optimal use of biomarkers for diagnosis as a complement to and according to clinical context.
• Global score of vitamin B_12_ deficiency probability based on the addition of both weighted clinical outcomes, biomarkers, and clinical context.
• Precision nutrition in vitamin B_12_ deficiency, with consideration of all clinical contexts, treatment options, and follow-up.
• Optimal use of biomarkers for follow-up in complementing and aligning with clinical context.

In conclusion, there is a need to develop forthcoming guidelines to formally standardize the diagnosis, etiologic work-up, and management of vitamin B_12_ deficiency across diverse health care settings. By integrating clinical and biomarker assessment and cause-specific treatment pathways, we aim to reduce both underdiagnosis and overdiagnosis, ultimately improving patient outcomes worldwide. Guidelines for children and adolescents and for pregnant/lactating women will be developed separately, reflecting the distinct epidemiology, diagnosis and management in these key populations.

## Author contributions

The authors’ responsibilities were as follows– J-LG, JLF, DHA: conceptualization, methodology, writing—original draft, and final content; LHA, EA, AMD, AG, RG, R-MGR, LH, DH, JBM, AM, JWM, MM, EN, HM, JPO, EQ, DR, HR, WR, SS, MW, CY: methodology and writing—review and editing; J-LG, JLF: project administration, resources, and supervision; J-LG, JLF, DHA, LHA, EA, AMD, AG, RG, R-MGR, LH, DH, JBM, AM, JWM, MM, EN, HM, JPO, EQ, DR, HR, WR, SS, MW, CY: review and editing; and all authors: read and approved the final manuscript.

## Declaration of AI and AI-Assisted Technologies in the Writing Process

The authors declare that no generative AI or AI-assisted technologies were used in the writing of this manuscript.

## Funding

The authors reported no funding received for this study.

## Conflict of interest

J-LG and MM acted as consultants for Italfarmaco in 2024. All other authors report no conflicts of interest

## References

[1] Green R., Allen L.H., Bjørke-Monsen A.L., Brito A., Guéant J.L., Miller J.W. (2017). Vitamin B_12_ deficiency. Nat. Rev. Dis. Primers..

[2] Guéant J.L., Guéant-Rodriguez R.M., Alpers D.H. (2022). Vitamin B12 absorption and malabsorption. Vitam. Horm..

[3] Guéant J.L., Guéant-Rodriguez R.M., Kosgei V.J., Coelho D. (2022). Causes and consequences of impaired methionine synthase activity in acquired and inherited disorders of vitamin B_12_ metabolism. Crit. Rev. Biochem. Mol. Biol..

[4] Levy J., Rodriguez-Guéant R.M., Oussalah A., Jeannesson E., Wahl D., Ziuly S. (2021). Cardiovascular manifestations of intermediate and major hyperhomocysteinemia due to vitamin B12 and folate deficiency and/or inherited disorders of one-carbon metabolism: a 3.5-year retrospective cross-sectional study of consecutive patients. Am. J. Clin. Nutr..

[5] Green R., Miller J.W. (2022). Vitamin B12 deficiency. Vitam. Horm..

[6] Sahu P., Thippeswamy H., Chaturvedi S.K. (2022). Neuropsychiatric manifestations in vitamin B12 deficiency. Vitam. Horm..

[7] Matmat K., Hassan Z., Pourié G., Atlasi Y., Seal S.V., Jeandel M. (2026). SIRT1 mediates brain metabolic and developmental consequences of methionine synthase deficiency in inborn errors of cobalamin metabolism, Cell. Rep. Med.

[8] Olivieri G., Bordugo A., Burnyte B., Costetti A., Couce M.L., Diogo L. (2026). First revision of the guidelines for the diagnosis and management of remethylation disorders. J. Inherit. Metab. Dis..

[9] Yetley E.A., Pfeiffer C.M., Phinney K.W., Bailey R.L., Blackmore S., Bock J.L. (2011). Biomarkers of vitamin B-12 status in NHANES: a roundtable summary. Am. J. Clin. Nutr..

[10] Allen L.H., Miller J.W., de Groot L., Rosenberg I.H., Smith A.D., Refsum H. (2018). Biomarkers of nutrition for development (BOND): vitamin B-12 review. J. Nutr..

[11] Sands T., Jawed A., Stevenson E., Smith M., Jawaid I. (2024). Guideline Committee, Vitamin B_12_ deficiency: NICE guideline summary. BMJ.

[12] Obeid R., Andrès E., Češka R., Hooshmand B., Guéant-Rodriguez R.M., Prada G.I. (2024). Diagnosis, treatment and long-term management of vitamin B12 deficiency in adults: a Delphi Expert consensus. J. Clin. Med..

[13] Bailey R.L., Carmel R., Green R., Pfeiffer C.M., Cogswell M.E., Osterloh J.D. (2011). Monitoring of vitamin B-12 nutritional status in the United States by using plasma methylmalonic acid and serum vitamin B-12. Am. J. Clin. Nutr..

[14] Stabler S.P. (2013). Vitamin B12 deficiency. N. Engl. J. Med..

[15] Wiedemann A., Oussalah A., Lamireau N., Théron M., Julien M., Mergnac J.P. (2022). Clinical, phenotypic and genetic landscape of case reports with genetically proven inherited disorders of vitamin B12 metabolism: a meta-analysis. Cell. Rep. Med..

[16] MacFarlane A.J. (2021). Hyperhomocysteinemia in patients with cardiovascular manifestations: to treat or not to treat. Am. J. Clin. Nutr..

[17] Lenti M.V., Miceli E., Vanoli A., Di Sabatino A. (2026). Autoimmune gastritis: emerging insights and clinical management. Nat. Rev. Gastroenterol. Hepatol..

[18] Yoo S., Montazeri A., Bennett D., Bo Y., Chen P., Duthie S. (2026). Folate and global health umbrella review series, part 1: methodological framework and syntheses on anaemia and neural tube defects. J. Glob. Health.

[19] Nakao S., Nakaseko C., Ishii T., Terai K., Shimizu N. (2025). Vitamin B6 deficiency may not always present as microcytic hypochromic anemia. Cureus.

[20] Alvarez M., Poveda S., Cisneros A., Parra D., Luna M., Rincón O. (2026). B vitamin deficiencies and associated neuropathies. Curr. Nutr. Rep..

[21] Andrès E., Zulfiqar A.A., Serraj K., Vogel T., Kaltenbach G. (2018). Systematic review and pragmatic clinical approach to oral and nasal vitamin B12 (cobalamin) treatment in patients with vitamin B12 deficiency related to gastrointestinal disorders. J. Clin. Med..

[22] Huber J., Hawkes J.E., Powell D.L. (2016). Cobalt-induced contact urticaria presenting as chronic urticaria due to intramuscular vitamin B12 injections. Dermatitis.

[23] Abadeh A., Herman S.M., Abdalian R. (2023). The prevalence of gastrointestinal symptoms and cobalamin deficiency in patients with chronic urticaria. Allergy Asthma Clin. Immunol..

[24] Liu K., Yang Z., Lu X., Zheng B., Wu S., Kang J. (2024). The origin of vitamin B12 levels and risk of all-cause, cardiovascular and cancer specific mortality: a systematic review and dose-response meta-analysis. Arch. Gerontol. Geriatr..

[25] Flores-Guerrero J.L., Minović I., Groothof D., Gruppen E.G., Riphagen I.J., Kootstra-Ros J. (2020). Association of plasma concentration of vitamin B12 with all-cause mortality in the general population in the Netherlands. JAMA Netw. Open.

[26] Wolffenbuttel B.H., Heiner-Fokkema M.R., Green R., Gans R.O. (2020). Relationship between serum B12 concentrations and mortality: experience in NHANES. BMC Med.

[27] Hashash J.G., Elkins J., Lewis J.D., Binion D.G. (2024). AGA clinical practice update on diet and nutritional therapies in patients with inflammatory bowel disease: expert review. Gastroenterology.

[28] Mahawar K.K., Reid A., Graham Y., Callejas-Diaz L., Parmar C., Carr W.R. (2018). Oral vitamin B_12_ supplementation after Roux-en-Y gastric bypass: a systematic review. Obes. Surg..

[29] Li Z., Gueant-Rodriguez R.M., Quilliot D., Sirveaux M.A., Meyre D., Gueant J.L. (2017). Folate and vitamin B12 status is associated with insulin resistance and metabolic syndrome in morbid obesity. Clin. Nutr..

[30] Antoine D., Li Z., Quilliot D., Sirveaux M.A., Meyre D., Mangeon A. (2021). Medium term post-bariatric surgery deficit of vitamin B12 is predicted by deficit at time of surgery. Clin. Nutr..

[31] Kornerup L.S., Hvas C.L., Abild C.B., Richelsen B., Nexo E. (2019). Early changes in vitamin B12 uptake and biomarker status following Roux-en-Y gastric bypass and sleeve gastrectomy. Clin. Nutr..

[32] Murphy M.M., Molloy A.M., Ueland P.M., Fernandez-Ballart J.D., Schneede J., Arija V. (2007). Longitudinal study of the effect of pregnancy on maternal and fetal cobalamin status in healthy women and their offspring. J. Nutr..

[33] Greibe E., Andreasen B.H., Lildballe D.L., Morkbak A.L., Hvas A.M., Nexo E. (2011). Uptake of cobalamin and markers of cobalamin status: a longitudinal study of healthy pregnant women. Clin. Chem. Lab. Med..

[34] Dib M.J., Gumban-Marasigan M., Yoxall R., Andrew T., Harrington D.J., Sobczyńska-Malefora A. (2022). Evaluating the diagnostic value of a combined indicator of vitamin B_12_ status (cB_12_) throughout pregnancy. Front. Nutr..

[35] Santos-Calderón L.A., Cavallé-Busquets P., Ramos-Rodríguez C., Grifoll C., Rojas-Gómez A., Ballesteros M. (2024). Folate and cobalamin status, indicators, modulators, interactions, and reference ranges from early pregnancy until birth: the Reus-Tarragona birth cohort study. Am. J. Clin. Nutr..

[36] Mathew A.R., Selita E., Regano C., Bianco C., Corsetti V., Cavallucci V. (2026). Vitamin B12 and reproductive health: clinical insights, emerging mechanistic understanding, and nutritional aspects. Mol. Reprod. Dev..

[37] Zwierz M., Suprunowicz M., Mrozek K., Pietruszkiewicz J., Oracz A.J., Konarzewska B. (2025). Vitamin B12 and autism spectrum disorder: a review of current evidence. Nutrients.

[38] Nie L., Liu X., Li X., Ren Z., Cheng X., Wu Y. (2025). Beyond folate: the emerging role of maternal vitamin B12 in neural tube development. Nutrients.

[39] Sukumar N., Rafnsson S.B., Kandala N.B., Bhopal R., Yajnik C.S., Saravanan P. (2016). Prevalence of vitamin B-12 insufficiency during pregnancy and its effect on offspring birth weight: a systematic review and meta-analysis. Am. J. Clin. Nutr..

[40] Sobczyńska-Malefora A., Yajnik C.S., Harrington D.J., Hitman G.A., Finer S. (2022). Vitamin B12 and folate markers are associated with insulin resistance during the third trimester of pregnancy in South Asian women, living in the United Kingdom, with gestational diabetes and normal glucose tolerance. J. Nutr..

[41] Oussalah A., Levy J., Berthezène C., Alpers D.H., Guéant J.L. (2020). Health outcomes associated with vegetarian diets: an umbrella review of systematic reviews and meta-analyses. Clin. Nutr..

